# Effect of Admixtures on Time-Dependent Gas Permeability of Concrete and Correlation with Its Microstructure Parameters

**DOI:** 10.3390/ma19163389

**Published:** 2026-08-10

**Authors:** Jiandong Wang, Jingzong Xu, Shumeng Zhang, Yinghui Cao

**Affiliations:** 1 Campus Construction Office, Zhejiang University of Technology, Hangzhou 310014, China; wjd@zjut.edu.cn; 2 College of Civil Engineering, Zhejiang University of Technology, Hangzhou 310014, China; 221125060089@zjut.edu.cn (J.X.); 302024524033@zjut.edu.cn (S.Z.)

**Keywords:** natural tidal environment, admixture concrete, gas permeability, microstructure, time dependent

## Abstract

The long-term durability of concrete exposed to marine environments is governed by progressive changes in transport behavior and pore network characteristics. Concrete mixtures incorporating fly ash (FA), slag (SG), silica fume (SF), and basalt fiber (BF) were subjected to natural tidal exposure, after which gas permeability and microstructural evolution were systematically characterized using nuclear magnetic resonance (NMR). Temporal trends in gas permeability were assessed in parallel with porosity and pore size distribution (PSD) measurements. Correlation analysis was subsequently performed to identify the pore characteristics that predominantly control gas transport. All investigated admixtures reduced gas permeability, although their effectiveness varied with exposure duration. Silica fume exhibited the greatest reduction during the early exposure stage, whereas fly ash became increasingly effective over prolonged exposure. Basalt fiber also contributed to improved gas transport resistance, while slag showed a comparatively weaker influence under the investigated conditions. The incorporation of admixtures strengthened the relationships between gas permeability and pore structure parameters. Among the investigated pore characteristics, the fraction of pores within the range of 100–500 nm exhibited the strongest correlation with permeability evolution. Furthermore, critical pore diameter and median pore diameter proved to be more representative indicators of gas transport resistance than mean pore diameter.

## 1. Introduction

The long-term durability of reinforced concrete structures exposed to marine environments is largely governed by the ingress of aggressive species, particularly chloride ions, which migrate through the interconnected pore network and eventually initiate reinforcement corrosion [1,2], resulting in progressive deterioration of structural durability [3,4]. Various transport-related parameters have therefore been proposed to evaluate the resistance of concrete to aggressive media, including gas permeability [5,6], water permeability [4,6], and chloride diffusivity [7,8]. Water permeability tests generally require prolonged stabilization periods and are strongly affected by the degree of pore saturation [6]. Likewise, chloride diffusion measurements are complicated by the time-dependent chloride-binding capacity of cement hydration products [3,9,10]. By comparison, gas permeability testing provides a rapid and practical means of assessing the connectivity of the pore system while avoiding the complexities associated with liquid–solid interactions [11,12]. Consequently, gas permeability has become one of the most widely adopted indicators for evaluating the durability of cement-based materials [5,11,13].

Supplementary cementitious materials and chemical admixtures are widely recognized as effective approaches for improving the transport resistance of concrete through pore structure refinement [12,13,14,15,16,17,18,19]. Existing studies have demonstrated that the permeability reduction achieved by these materials depends strongly on their composition, replacement level, and curing conditions. The incorporation of nano-SiO_2_ has been demonstrated to refine the microstructure of cement-based materials by promoting matrix densification and modifying hydration products, thereby improving transport resistance [13]. Silica fume also exhibits remarkable permeability reduction under relatively unfavorable curing conditions (20 °C and 65% RH), producing decreases of approximately 71% after 1 day and 87% after 365 days compared with ordinary concrete [14]. Similar improvements have been observed in blended cement systems, where secondary hydration reactions and the formation of additional hydration products contribute to pore refinement and reduced gas transport during long-term curing [7]. In contrast, comparative investigations indicate that concrete containing 50% slag generally exhibits higher gas permeability than mixtures incorporating 30% fly ash under identical curing conditions [15], suggesting that different supplementary cementitious materials contribute differently to permeability evolution.

The gradual reduction in concrete permeability is primarily attributed to the continuous formation of hydration products and the progressive refinement of the pore network. The rate of permeability evolution is influenced by multiple factors, including admixture composition [16], replacement level [20], exposure environment [12,17], curing regime, and the resulting pore connectivity characteristics of blended concrete [21]. Previous investigations consistently indicate that gas permeability decreases with curing age, although different supplementary cementitious materials exhibit distinct long-term performances. Ground granulated blast-furnace slag generally provides greater resistance to gas transport than fly ash under equivalent curing conditions [16], whereas increasing fly ash replacement leads to pronounced differences in permeability during hydration, with further reductions becoming evident after 90 days [20]. Curing conditions likewise exert considerable influence on permeability development. Variations in curing conditions can significantly influence air permeability by modifying hydration processes and pore connectivity within blended concrete systems [21]. Environmental variables, including chloride concentration, temperature, and electrochemical conditions, further modify chloride binding and ionic transport [10,22], thereby promoting pore refinement and increasing transport tortuosity. Similar trends have also been confirmed through long-term marine exposure investigations extending over 23 years [21].

Despite these advances, most available permeability data have been obtained from specimens cured under controlled laboratory conditions for relatively short durations, typically ranging from 90 to 180 days [12,13,20]. Although such studies provide valuable insights into permeability development, they cannot fully reproduce the complex hydration processes and fluctuating environmental actions experienced under natural exposure conditions [23]. Consequently, increasing attention has been directed toward long-term field exposure investigations, which provide a more realistic basis for evaluating permeability evolution throughout the service life of concrete structures [24].

Gas transport in concrete is fundamentally governed by the characteristics of the internal pore network, among which total porosity [13,24], pore size distribution [7,14], and pore connectivity [5,25] are generally considered the primary parameters controlling permeability. As hydration progresses and supplementary cementitious materials continue to react, the pore structure gradually becomes denser, resulting in simultaneous reductions in porosity and gas permeability [14,24]. Mineral admixtures facilitate the conversion of coarse capillary pores into finer pores through secondary hydration, whereas nano-SiO_2_ modification and fiber reinforcement further improve matrix compactness and the interfacial transition zone [18,19]. In addition, reductions in total porosity together with refinement of the most probable pore diameter have been closely associated with enhanced resistance to both gas transport and chloride ingress [13,25]. These findings indicate that permeability evolution is governed by the combined effects of multiple pore characteristics rather than by total porosity alone.

Pore structure characterization of cement-based materials commonly relies on various experimental techniques, including mercury intrusion porosimetry (MIP), gas adsorption, and microscopic methods, to evaluate pore size distribution and pore connectivity characteristics [26], and backscattered electron (BSE) imaging [27]. Although these techniques provide detailed quantitative and morphological information, sample preparation procedures such as drying may alter the original moisture state and pore structure characteristics to some extent [26,27]. Furthermore, permeability estimations based on these methods commonly rely on empirical models, including the Katz–Thompson [28] and Garboczi [29] relationships, which infer transport properties indirectly rather than measuring them directly. Low-field nuclear magnetic resonance (NMR), by contrast, enables non-destructive characterization of water-filled pore structures without invasive pretreatment, thereby preserving the integrity of the cementitious matrix [6,30]. Its capability to simultaneously determine porosity, pore size distribution, and characteristic pore diameters makes it particularly suitable for monitoring long-term microstructural evolution under natural exposure conditions [30].

In light of the above considerations, the present study is designed to systematically investigate the time-dependent gas permeability and microstructural evolution of concrete containing four types of admixtures—fly ash (FA), slag (SG), silica fume (SF), and basalt fiber (BF)—under natural tidal exposure over a period of up to 1160 days. Gas permeability coefficients are measured at eight discrete exposure ages using a custom-built apparatus based on the RILEM Cembureau method. Concurrently, pore structure parameters, including total porosity, pore size distribution, critical pore diameter, median pore diameter, and mean pore diameter, are characterized via low-field NMR. Specifically, this study aims to: (1) to quantify the temporal evolution of gas permeability under cyclic wet–dry tidal conditions and to compare the long-term efficiency of each admixture; (2) to delineate the time-dependent pore refinement effects and the corresponding evolution of multi-scale pore structure; (3) to establish quantitative correlations between gas permeability and key pore indicators, with particular emphasis on identifying the pore size fraction most sensitive to gas transport; and (4) to evaluate the applicability of an existing time-dependent permeability prediction model under natural tidal conditions. The ultimate goal is to provide a mechanistic understanding of how admixture incorporation, pore structure evolution, and time-varying permeability are coupled, thereby supporting durability-based design of concrete structures in coastal environments.

## 2. Experimental Methods

### 2.1. Raw Materials and Mixture Proportions of Concrete

Concrete mixtures were prepared using ordinary constituents commonly employed in hydraulic engineering applications. River sand with a fineness modulus of 2.4 and an apparent density of 2600 kg/m^3^ was used as the fine aggregate, while crushed gravel with a maximum particle size of 20 mm and an apparent density of 2700 kg/m^3^ served as the coarse aggregate. Ordinary tap water was used for both concrete mixing and standard curing. The binder consisted of Qian-Chao composite Portland cement (P.C. 32.5). Four different admixtures were incorporated in this study, including Class I fly ash (FA), S95 ground granulated blast-furnace slag (SG), silica fume (SF), and chopped basalt fiber (BF). The fly ash possesses a specific surface area of 540 kg/m^3^, whereas the corresponding values for slag and silica fume are 450 m^2^/kg and 18,954 m^2^/kg, respectively. The silica fume exhibits a loss on ignition of 2.28%. The basalt fibers have a filament diameter of 17–20 μm, a length of 10–20 mm, a tensile strength ranging from 2800 to 3800 MPa, and an elastic modulus between 100 and 110 GPa.

The chemical compositions of the cement and the four admixtures are summarized in Table 1.

To evaluate the influence of different admixtures on the long-term evolution of gas permeability and pore structure under natural tidal exposure, six concrete mixtures were designed. A constant water-to-binder ratio of 0.50 and a sand ratio of 32% were adopted for all mixtures to eliminate the influence of mixture proportion variations. Fly ash, slag, and silica fume were introduced as partial cement replacements on an equal-mass basis, whereas basalt fibers were added directly without replacing cement. The detailed mixture proportions together with the corresponding 28-day compressive strengths are presented in Table 2.

### 2.2. Test Environment

**Figure 1:**
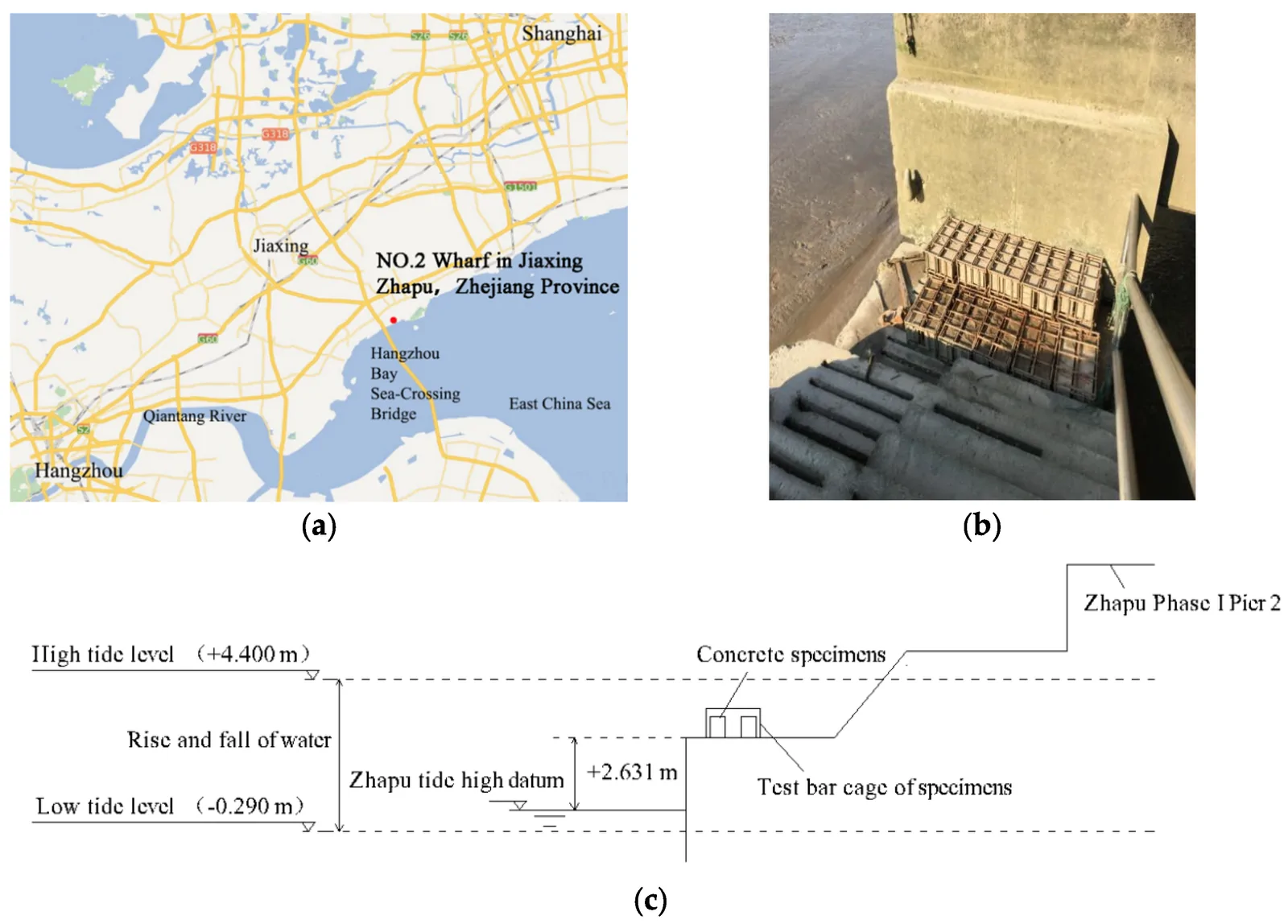
Geographical position of the exposure test under the natural tidal environment and the location of the site photos and components. (**a**) Geographical location of field trials. (**b**) Photos of some components of the field test (low tide). (**c**) Schematic diagram of the location of the concrete test piece for the field test.

The test site is located at No. 2 pier of Zhapu Port, Zhejiang Province, China. The port is located in the southern wing of the Yangtze River Delta and the north bank of Hangzhou Bay in Zhejiang Province. The location of field test is shown in Figure 1.

**Figure 2:**
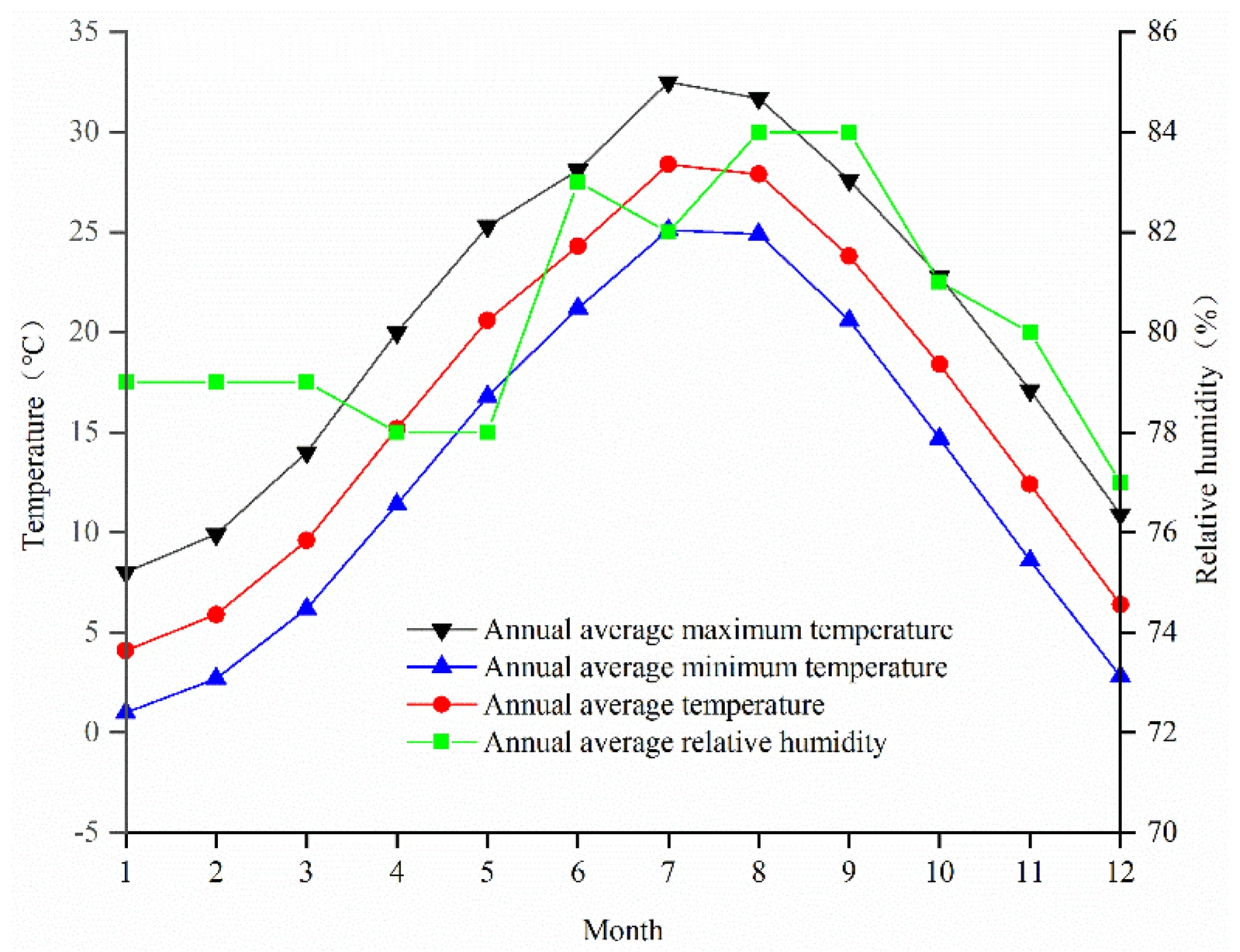
Multi-year monthly average temperature and relative humidity (1981~2019).

According to the field meteorological and hydrological data in the test location, the average drying time for this location is about 22 h per day, and the average immersion time is about 2 h, that is, the average dry–wet cycle time ratio is 11:1. The multi-year average temperature is 15.6 °C, and the multi-year monthly average temperature and relative humidity are shown in Figure 2 [31].

### 2.3. Preparation of Specimens

Concrete specimens were fabricated in accordance with the Chinese standard Test Code for Hydraulic Concrete (SL 352–2019 [32]). For each mixture and exposure age, two prism specimens measuring 150 mm × 150 mm × 550 mm and three cube specimens with dimensions of 150 mm × 150 mm × 150 mm were cast. After standard curing for 28 days under a temperature of 20 ± 5 °C and a relative humidity of no less than 95%, the compressive strength of each mixture was determined, and the results are listed in Table 2.

Eight exposure durations were considered in the present investigation, namely 0, 120, 240, 360, 520, 680, 840, and 1160 days, where the 0-day condition corresponds to specimens immediately tested after the initial 28-day standard curing period. All concrete specimens were prepared in a single batch to eliminate variability arising from material or casting differences. Upon completion of the standard curing period, the specimens were installed in specially fabricated reinforcing cages and subsequently transferred to the tidal exposure site shown in Figure 1.

**Figure 3:**
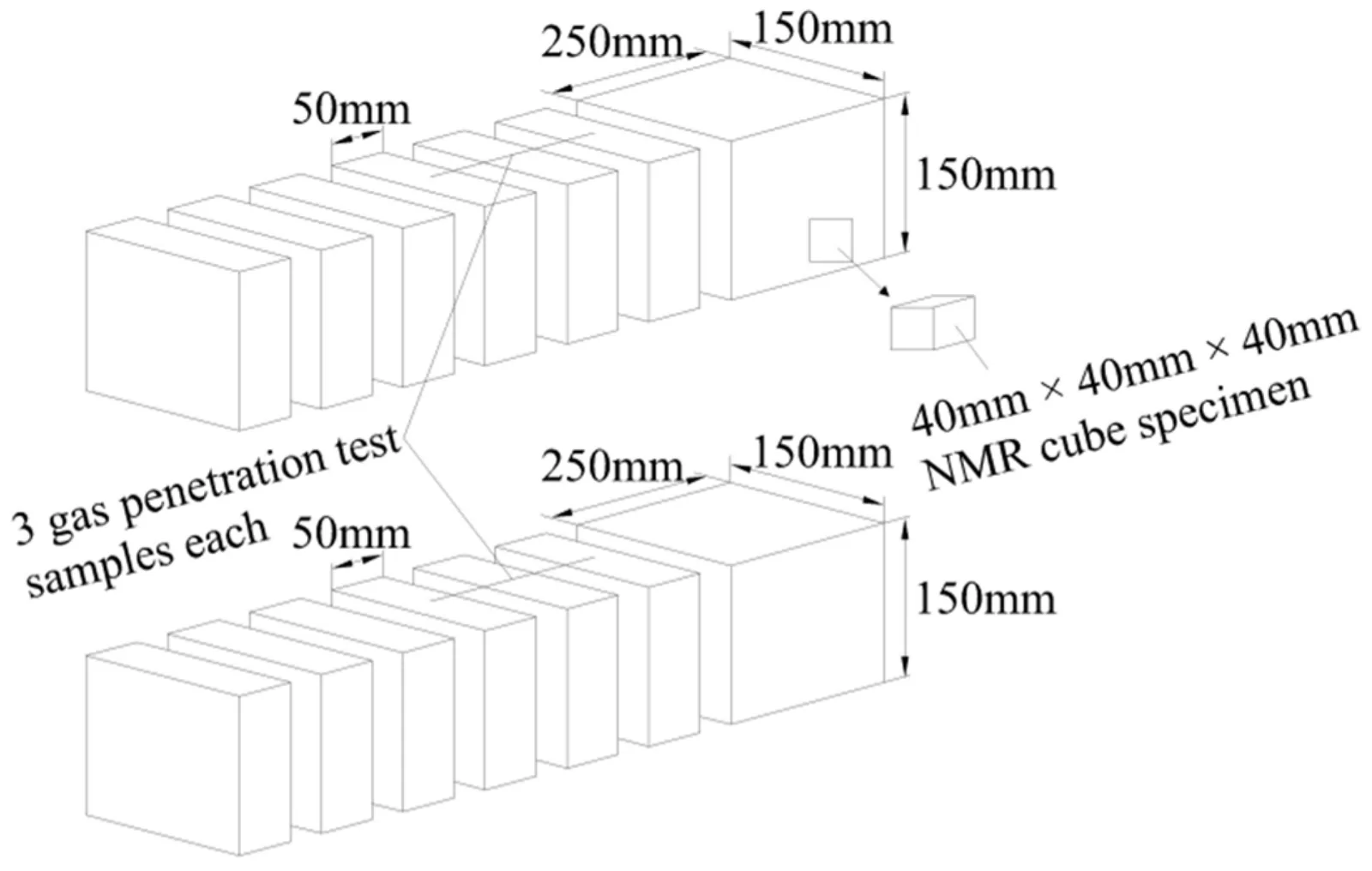
Cutting schematic diagram for gas permeability and NMR tests.

After reaching each designated exposure age, the specimens were transported to the laboratory for subsequent testing. Representative samples for gas permeability and NMR measurements were obtained using an automatic diamond saw. The dimensions of the gas permeability specimens and NMR specimens were 150 mm × 150 mm × 50 mm and 40 mm × 40 mm × 40 mm, respectively, as illustrated in Figure 3. To improve the statistical reliability of the permeability measurements and minimize the influence of material heterogeneity, six independent specimens were prepared for each gas permeability test.

### 2.4. Test Procedure

#### 2.4.1. Test Method of Gas Permeability Coefficient

**Figure 4:**
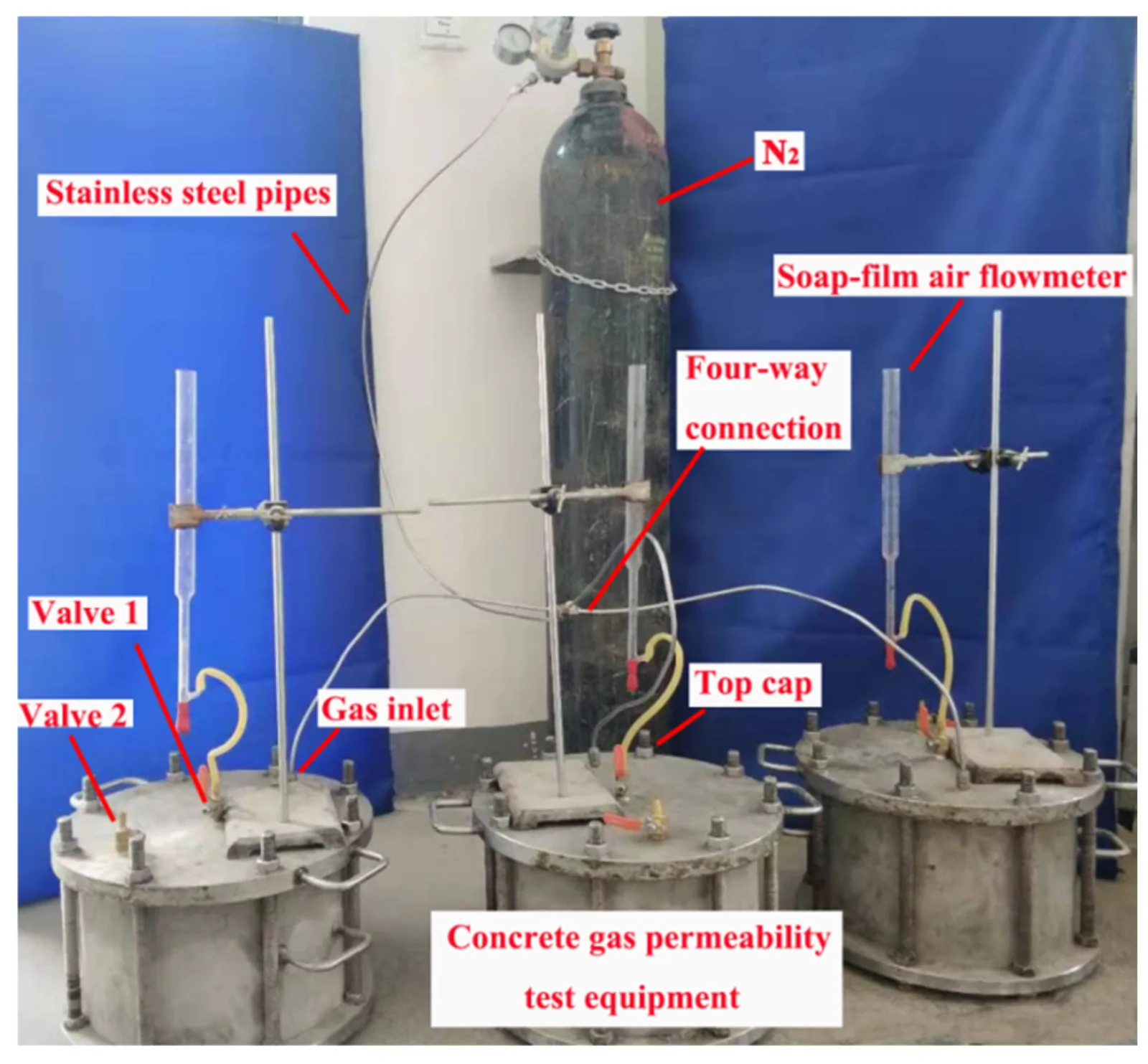
Concrete gas permeability test device.

Gas permeability was evaluated using a custom-designed apparatus developed on the basis of the RILEM Cembureau recommendation [33]. The device allows multiple concrete specimens to be tested simultaneously, thereby improving testing efficiency and reducing experimental variability. A schematic illustration of the apparatus is presented in Figure 4.

Prior to testing, all specimens were oven-dried at 105 ± 5 °C for 48 h to remove moisture from the pore system and were subsequently cooled to room temperature. High-purity nitrogen (N_2_) was employed as the permeating gas. During testing, one surface of the specimen was maintained under atmospheric pressure, while a constant inlet pressure of 0.3 MPa was applied to the opposite face. After approximately 30 min, the gas flow reached a steady state, at which point the flow rate was measured.

Because of the relatively low permeability of concrete, the gas flow rate was determined using a soap-film flow meter. The travel time required for the soap bubble to move from 0 to 50 mL was recorded, and the average of three consecutive measurements was adopted to calculate the volumetric flow rate.

According to Darcy’s law [34], the intrinsic gas permeability coefficient is expressed as

(1) $$Q=\frac{-KA}{\mu }\frac{dP}{dZ}$$

where Q denotes the volumetric gas flow rate (m^3^/s), K is the intrinsic permeability coefficient (m^2^), A represents the specimen cross-sectional area (m^2^), μ is the dynamic viscosity of the permeating gas, and dp/dz is the pressure gradient (N/m^3^).

Considering the compressibility of gas, the measured permeability was corrected using the relationship proposed in Ref. [35]:

(2) $$K_{g}=\frac{V}{t}\frac{h}{A}\mu_{g}\frac{2P_{a}}{\left(P^{2}-P_{a}^{2}\right)}$$

where $\mu_{g}$ is the dynamic viscosity of nitrogen at the experimental temperature, $s\cdot N\cdot m^{-2}$ ; V is the total volume flow of nitrogen, L; *h* is the thickness of the sample at the flow direction, m; A is the cross-sectional area through nitrogen, m^2^; Ρ is the inlet pressure, N/m^2^; ${Ρ}_{a}$ is the atmospheric pressure, N/m^2^.

For the present testing configuration, the effective flow area was 0.064 m^2^. The dynamic viscosity of nitrogen was calculated as a function of temperature according to Ref. [36]:

(3) $$\mu_{g}=16.68529+0.04468T$$

where T is the testing temperature, °C.

#### 2.4.2. NMR Test Method

The pore structure of concrete was characterized using a low-field nuclear magnetic resonance (NMR) analyzer (MesoMR23-60H-I, Niumag Electric Corporation, Suzhou, China) [30]. Prior to testing, all specimens were subjected to a saturation treatment to ensure that the pore system was completely filled with water. The preparation procedure consisted of three steps:

1. Surface dust and loose particles were carefully removed using a soft brush;
2. The specimens were vacuum-saturated in distilled water at 0.1 MPa for 10 h;
3. After saturation, excess surface water was removed, the specimens were wrapped with plastic film to prevent moisture loss, and then immediately placed in the NMR probe for measurement.

Low-field NMR characterizes the pore structure by detecting the transverse relaxation behavior of hydrogen nuclei within pore water. Under the fast-exchange assumption, the transverse relaxation time ( $T_{2}$ ) of a water-filled pore is proportional to its surface-to-volume ratio, allowing pore size information to be obtained without damaging the specimen. Consequently, the distribution can be converted into pore size distribution (PSD), porosity, and characteristic pore diameters, which are closely associated with transport behavior in cement-based materials [24,28].

The relationship between pore radius and transverse relaxation time is expressed as

(4) $$\frac{1}{T_{2}}\approx \rho_{2}\frac{S}{V}$$

where $\rho_{2}$ represents the surface relaxation rate (ms), and S/V is the pore surface-to-volume ratio (nm^−1^).

## 3. Results and Discussion

### 3.1. Time-Dependent Gas Permeability

Outlying data were first screened using the Grubbs test [37]. After excluding invalid measurements, the gas permeability coefficient at each exposure age was determined from the average of no fewer than four valid specimens. The resulting permeability coefficients are summarized in Table 3.

Table 3 demonstrates a continuous reduction in gas permeability for all concrete mixtures throughout the 1160-day tidal exposure period, indicating that resistance to gas transport progressively improves with time. This general trend reflects the gradual refinement of the pore network during long-term hydration and environmental exposure. Similar improvements in transport resistance have been reported for blended cement systems due to secondary hydration and pore structure refinement [7,14,16].

The evolution of the individual mixtures also exhibits noticeable differences. Mixture A3 achieves the lowest permeability after approximately 360 days and subsequently maintains values comparable to those of A2. By comparison, the permeability of slag-modified concrete (A4) remains close to that of the plain concrete (A1) over the entire exposure period, suggesting that the relatively low slag replacement adopted in the present study provides only a limited contribution to permeability refinement [16]. Silica-fume concrete (A5) exhibits the smallest permeability coefficients before 240 days, highlighting its pronounced effectiveness during the early exposure stage. However, the reduction rate becomes much less significant after prolonged exposure. Basalt-fiber concrete (A6) displays a different evolution pattern, showing higher permeability than the control mixture during the first 120 days but progressively outperforming the control after 240 days, which indicates that the beneficial influence of basalt fibers mainly develops during long-term exposure.

**Figure 5:**
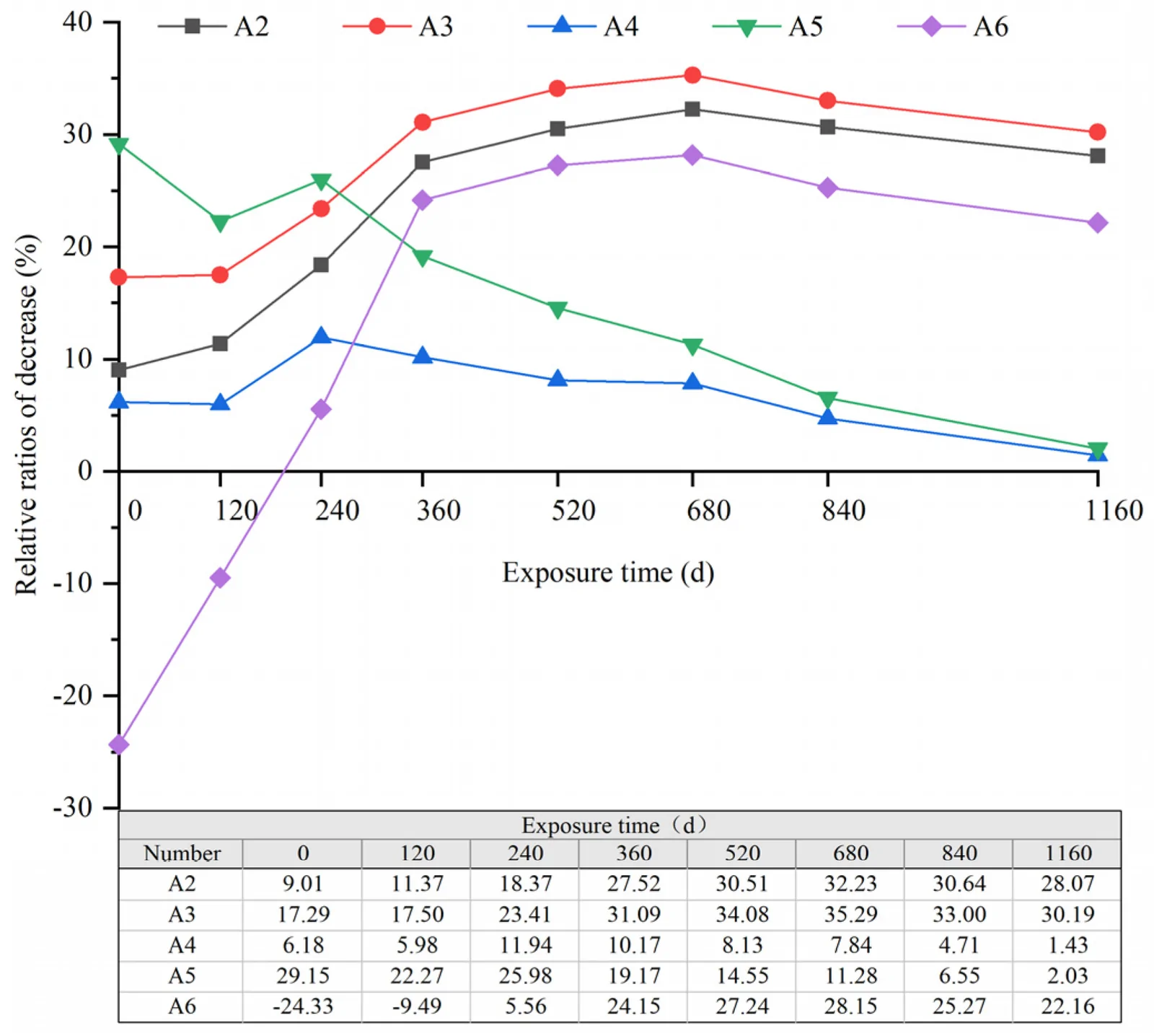
Reducing degree of gas permeability coefficient of concrete with different admixtures.

To quantitatively compare the effectiveness of different admixtures, the permeability reduction ratio, α, is introduced as

(5) $$\alpha =\frac{K_{g.0}(t)-K_{g.i}(t)}{K_{g.0}(t)}\times 100\%$$

where $K_{g.0}\left(t\right)$ and $K_{g.i}\left(t\right)$ denote the gas permeability coefficients of the plain concrete and the concrete incorporating the ith admixture at the same exposure age, respectively. The calculated values of α are presented in Figure 5.

Figure 5 indicates that all four admixture systems reduce gas permeability throughout the exposure period, although their relative efficiencies evolve differently with time. Such differences primarily arise from variations in hydration kinetics, pozzolanic activity, and the mechanisms by which each admixture modifies the pore structure [14,16].

For the fly ash mixtures (A2 and A3), the permeability reduction becomes increasingly pronounced with exposure time before gradually approaching a stable level after approximately 680 days. At this stage, mixture A3 exhibits a 35.29% lower permeability than the reference concrete A1. This evolution reflects the changing role of fly ash during hydration. Initially, permeability improvement is mainly associated with the micro-filler effect, whereby fine fly ash particles occupy voids between cement grains. As hydration proceeds, secondary pozzolanic reactions consume calcium hydroxide and generate additional C-S-H gel, producing continuous pore refinement and further restricting gas transport [16,38]. The gradually diminishing reduction rate after long-term exposure suggests that the pore structure has approached a relatively stable condition.

The influence of slag is comparatively limited. Throughout the entire exposure period, mixture A4 exhibits only a moderate reduction in permeability relative to the control concrete. Although a slight increase in the reduction ratio is observed before 240 days, little additional improvement occurs thereafter. This relatively weak response is most likely associated with the low slag replacement level (5%), which restricts both the filler effect and the subsequent pozzolanic reaction, thereby limiting its contribution to long-term pore refinement [16].

Silica fume produces the most significant improvement during the early exposure stage. Before 240 days, A5 exhibits the highest permeability reduction among all mixtures, with α values reaching 29.15% at the beginning of exposure and remaining above 25% after 240 days. The rapid improvement can be attributed to the ultrafine particle size and extremely high pozzolanic reactivity of silica fume, which accelerate the formation of secondary C–S–H gel while simultaneously densifying the interfacial transition zone (ITZ) [39]. Because only 5% silica fume is incorporated, however, the available reactive particles are consumed relatively quickly. Consequently, the rate of permeability reduction decreases noticeably during later exposure, leading to only limited further improvement after approximately 360 days.

Unlike the mineral admixtures, basalt fiber affects permeability mainly through modifications to the internal cracking behavior rather than through secondary hydration. Before 120 days, A6 exhibits higher permeability than the plain concrete because randomly distributed fibers introduce additional interfacial transition zones and increase local heterogeneity within the matrix [40,41]. As hydration continues, the bond between fibers and the surrounding cement paste becomes progressively stronger, while the fibers effectively restrain shrinkage-induced microcracking and delay crack propagation. These mechanisms gradually outweigh the initial adverse effect of the fiber interfaces, allowing the permeability of A6 to decrease below that of the control concrete after approximately 240 days. By 680 days, the permeability reduction reaches 28.15%, demonstrating that basalt fibers contribute primarily to long-term rather than early-age transport resistance.

### 3.2. Influence of Admixture on the Time-Varying Gas Permeability of Concrete

To characterize the long-term evolution of gas permeability, the empirical relationship proposed by Zhang et al. [25] was employed to fit the experimental results:

(6) $$K_{g}\left(t\right)=K_{g_{0}}{\left(\frac{t_{0}}{t+t_{0}}\right)}^{m_{g}}$$

where $K_{g}\left(t\right)$ is the gas permeability coefficient at exposure age t , $K_{g_{0}}$ denotes the corresponding permeability coefficient at the reference curing age $t_{0}$ , and $m_{g}$ represents the age reduction factor describing the rate of permeability evolution. In the present study, $t_{0}$ was taken as 28 days according to the standard curing period.

Using the permeability data listed in Table 3, values of the age reduction factor were obtained for different exposure intervals by fitting Equation (6). The corresponding fitting results together with the coefficients of determination are summarized in Table 4.

A clear dependence of the age reduction factor on both exposure duration and admixture type can be identified. Except for silica-fume concrete (A5), all mixtures exhibit a gradual increase in $m_{g}$ as exposure proceeds, indicating that permeability reduction becomes progressively more pronounced during long-term tidal exposure. Meanwhile, the incremental increase in $m_{g}$ becomes smaller at later ages, suggesting that the microstructure gradually approaches a relatively stable state as hydration and pore refinement continue.

Fly ash mixtures (A2 and A3) consistently present larger age reduction factors than the plain concrete (A1), demonstrating that fly ash enhances the long-term rate of permeability reduction. Although increasing the fly ash replacement from 20% to 30% slightly decreases the fitted value of $m_{g}$ , both mixtures exhibit a similar evolutionary trend, with the growth of the reduction factor becoming increasingly moderate after approximately 840 days. This behavior reflects the gradual exhaustion of the pozzolanic reaction as available calcium hydroxide decreases, causing the refinement of the pore structure to slow at later ages. Comparable observations have been reported by Khan et al. [42], who found that the influence of fly ash on oxygen permeability becomes increasingly evident after prolonged curing. Considering that the present specimens experienced continuous wetting-drying cycles under natural tidal conditions rather than constant laboratory curing, stabilization of the age reduction factor after long-term exposure appears reasonable.

A different trend is observed for slag-modified concrete. Throughout the investigated period, the fitted $m_{g}$ values remain slightly higher than those of the control mixture and become nearly constant after about 680 days. This indicates that slag contributes to permeability reduction mainly during the earlier stages of exposure, after which its influence gradually diminishes. The relatively limited variation is consistent with the low slag replacement adopted in this study and agrees with the observations reported by Shi et al. [16].

Silica-fume concrete (A5) exhibits the smallest age reduction factor among all mixtures, with $m_{g}$ showing a slight downward trend as exposure time increases. Rather than indicating inferior durability, this behavior reflects the rapid development of permeability during the initial exposure stage. Because silica fume reacts quickly with calcium hydroxide and rapidly densifies the matrix, much of its beneficial effect is realized within the first few months of exposure. Once the available silica fume has largely participated in the pozzolanic reaction, subsequent reductions in permeability become relatively limited. This interpretation agrees well with the permeability evolution presented in Table 3 and is also consistent with previous findings reported by Hassan et al. [14].

Among all investigated mixtures, basalt-fiber concrete (A6) exhibits the highest age reduction factor. The relatively large $m_{g}$ value reflects the substantial difference between its early-age and long-term permeability behavior. Additional interfacial transition zones introduced by the fibers initially increase gas transport; however, continued hydration progressively strengthens the fiber-matrix interface and suppresses the initiation and propagation of microcracks. As these beneficial mechanisms become dominant, permeability decreases more rapidly than in the plain concrete, leading to the highest fitted age reduction factor [41].

**Figure 6:**
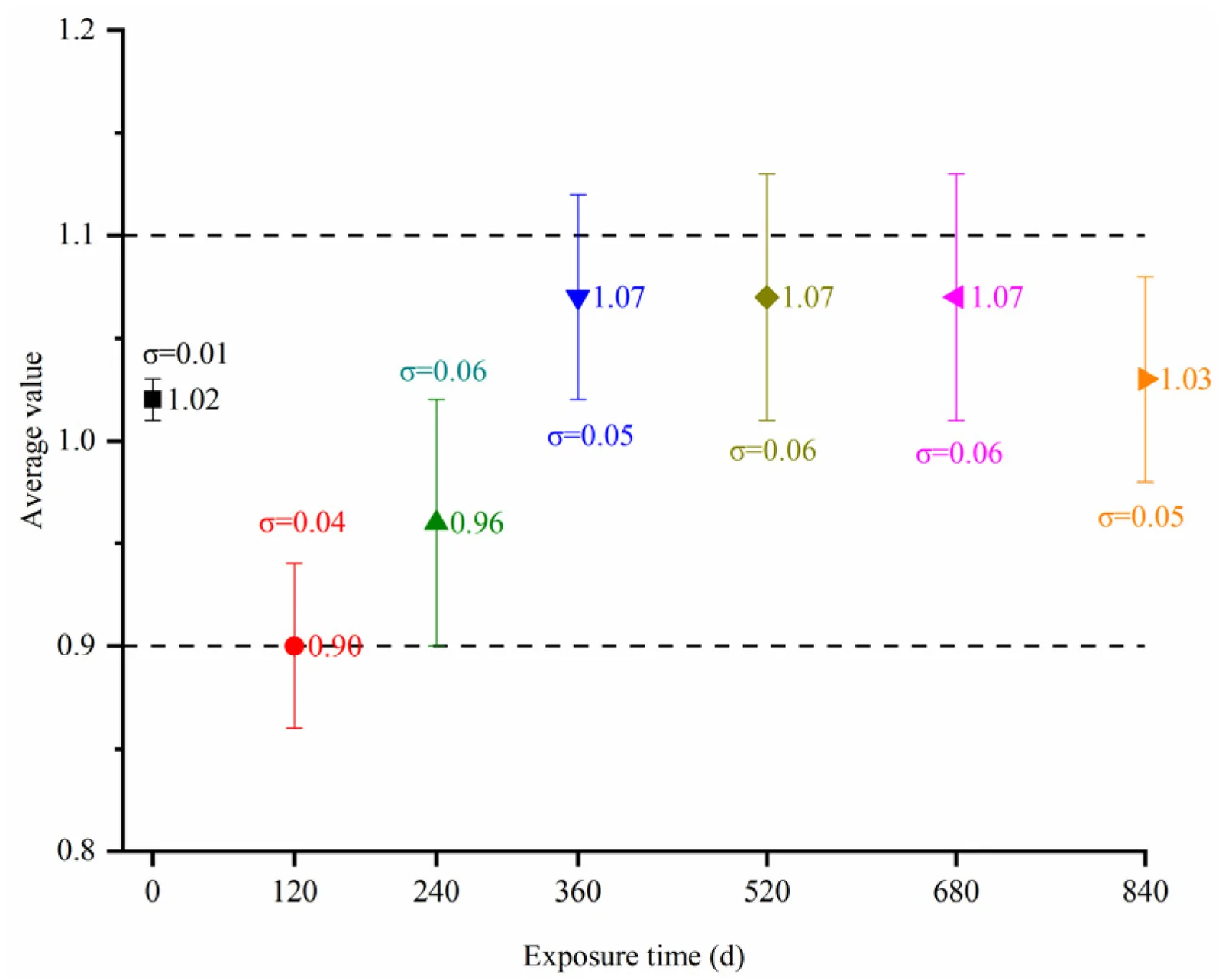
Average value and standard deviation of the ratio between the measured and predicted gas permeability coefficient.

To further evaluate the applicability of Equation (6) under natural marine exposure conditions, permeability data obtained up to 840 days were used to calibrate the model for each concrete mixture. The resulting predictions were subsequently compared with the corresponding experimental measurements, and the comparison is illustrated in Figure 6.

Overall, the predicted permeability coefficients agree closely with the measured values throughout the calibration period. For all exposure ages, the average ratio between prediction and experiment remains within the range of approximately 0.9–1.1, indicating that the fitted model satisfactorily reproduces the observed permeability evolution under tidal exposure.

The predictive capability of the calibrated model was further examined using the independent measurements obtained after 1160 days. A comparison between predicted and measured permeability coefficients is presented in Table 5. For every mixture, the relative prediction error remains below 10%, confirming that the fitted model maintains satisfactory accuracy even beyond the calibration period. These results demonstrate that the empirical relationship proposed by Zhang et al. [25] remains applicable for describing the long-term permeability evolution of concretes incorporating different mineral admixtures and basalt fibers under natural tidal exposure.

### 3.3. Microscopic Parameters and Its Time-Dependence of Test Concrete

#### 3.3.1. T_2_ Spectrum

**Figure 7:**
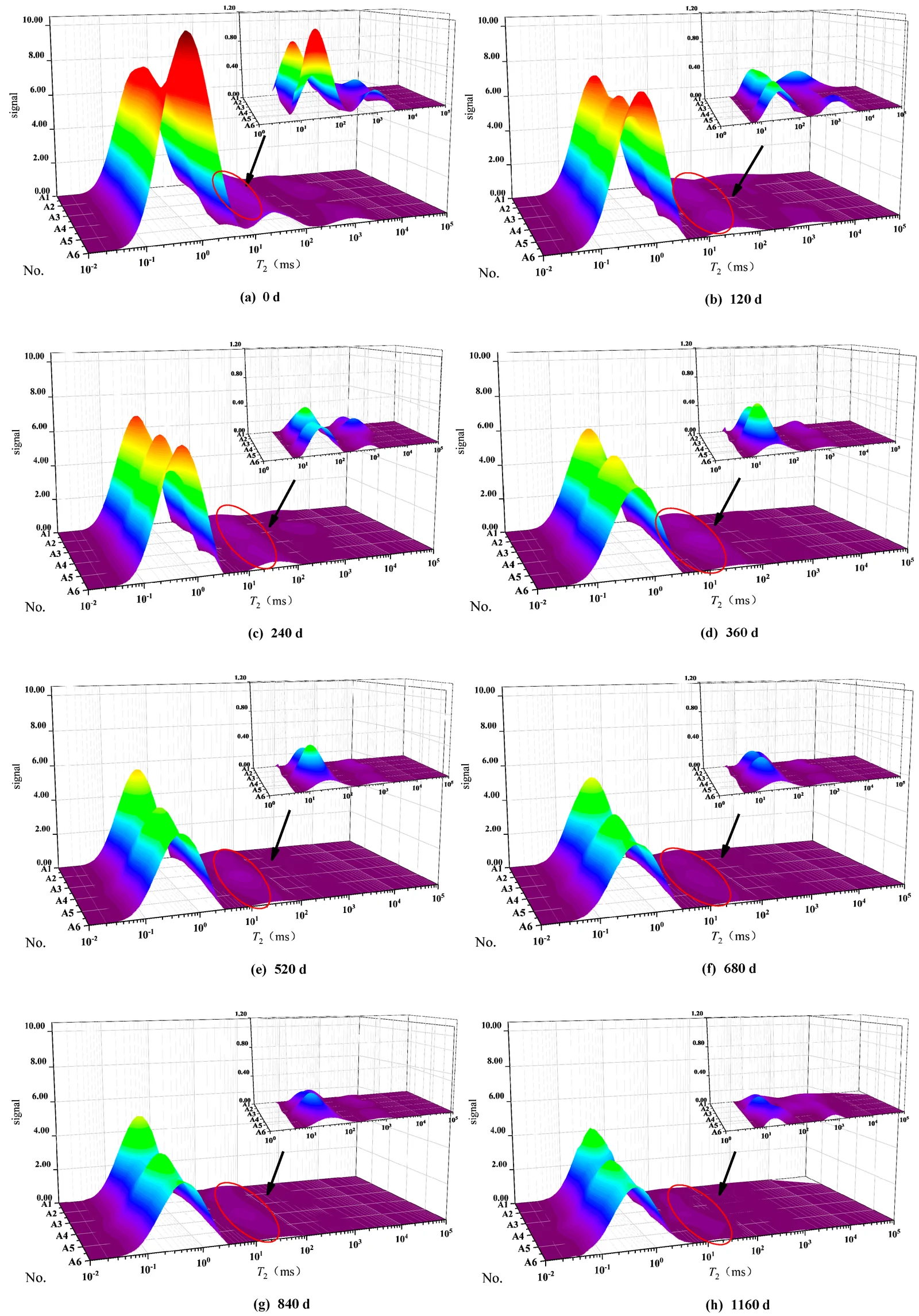
*T*_2_ spectrum of concrete at different exposure times.

The evolution of the pore system during long-term tidal exposure was further investigated using low-field NMR. The corresponding transverse relaxation time (*T*_2_) distributions for all concrete mixtures are presented in Figure 7. Because the transverse relaxation time is directly associated with the size of water-filled pores, the *T*_2_ spectrum provides valuable information regarding pore refinement, while the integrated signal area represents the total pore volume within the specimen [40].

Regardless of mixture composition, the overall intensity of the *T*_2_ spectra gradually decreases with increasing exposure time. This continuous attenuation indicates that the total volume of accessible pores becomes progressively smaller as hydration and secondary reactions proceed. During the early exposure stage (0–120 d), three distinct relaxation peaks are clearly identifiable, approximately corresponding to the ranges of 101–101 ms, 101–102 ms, and 102–104 ms. As exposure continues, the third peak gradually diminishes and becomes considerably flatter after approximately 240 days, suggesting that relatively large pores are continuously consumed or transformed into finer pore classes during long-term exposure.

In addition to the reduction in signal intensity, a systematic leftward shift in the dominant relaxation peaks can be observed for all admixture-modified concretes relative to the plain concrete. At an exposure age of 840 days, for example, the principal peak of mixture A1 is located at approximately 0.57 ms, whereas the corresponding peaks of all modified mixtures appear below 0.49 ms. Such a shift toward shorter relaxation times reflects progressive pore refinement and indicates that supplementary cementitious materials effectively promote the transformation of relatively coarse pores into finer pores during hydration [43].

Differences among admixture types become increasingly evident as exposure proceeds. Throughout most of the investigated period, the fly ash mixtures (A2 and A3) exhibit lower signal intensities than the control concrete within the relaxation range below 10^2^ ms, indicating a lower volume of effective transport pores. The difference becomes particularly pronounced after approximately 680 days, implying that the delayed pozzolanic reaction of fly ash continues to refine the pore structure during long-term exposure. By comparison, slag concrete (A4) exhibits the lowest signal intensity during the initial exposure period but shows relatively limited subsequent evolution, consistent with its comparatively modest influence on permeability development discussed in Section 3.1.

Silica-fume concrete (A5) displays the most significant reduction in signal intensity before 240 days, confirming that silica fume rapidly modifies the pore structure during the early stage of hydration. After this period, however, only minor changes are observed in the *T*_2_ spectrum, indicating that most of the pore refinement has already been completed. Basalt-fiber concrete (A6) exhibits a distinctly different evolution pattern. Before 120 days, its signal intensity exceeds that of the plain concrete over much of the relaxation range, reflecting the additional interfacial transition zones introduced by randomly distributed fibers. As hydration progresses, the signal gradually decreases below that of the control mixture, demonstrating that the beneficial influence of fibers on pore refinement becomes increasingly dominant during prolonged exposure.

Overall, the temporal evolution of the *T*_2_ spectra corresponds closely to the permeability development presented in Table 3. Mixtures exhibiting greater reductions in signal intensity and more pronounced shifts toward shorter relaxation times generally show lower gas permeability at the corresponding exposure ages. This consistency indicates that low-field NMR provides a reliable means of qualitatively evaluating the long-term evolution of transport properties through changes in pore structure [36].

#### 3.3.2. Total Porosity

The total porosity determined from the NMR measurements at different exposure ages is summarized in Table 6.

A continuous reduction in porosity is observed for all mixtures throughout the entire exposure period, indicating that the internal pore network becomes progressively denser under long-term tidal exposure. Although the reduction rate is relatively high during the early stage, it gradually decreases with increasing exposure time, suggesting that the hydration process and the associated pore refinement progressively approach equilibrium. This overall trend closely resembles the evolution of gas permeability presented in Table 3, implying that pore densification is one of the principal mechanisms governing the improvement of transport resistance.

Differences among admixture types become evident during the early exposure stage. Before 120 days, all modified concretes except A6 exhibit lower porosity than the plain concrete (A1), indicating that mineral admixtures are effective in accelerating matrix densification immediately after curing. In contrast, basalt-fiber concrete initially contains a larger pore volume because the introduction of fibers increases the number of interfacial transition zones and slightly disturbs the packing of the cementitious matrix [44].

Among all mixtures, silica-fume concrete (A5) exhibits the lowest porosity before approximately 240 days. This superior early-age performance results from the combined effects of ultrafine particle filling and rapid pozzolanic reactions, both of which promote the formation of additional C-S-H gel and effectively eliminate larger capillary pores [14]. However, the reduction in porosity becomes considerably less pronounced after prolonged exposure, indicating that the beneficial contribution of silica fume is largely realized during the initial hydration period.

The evolution of the fly ash mixtures differs from that of silica fume. Although A2 and A3 do not exhibit the smallest porosity during the early stage, their pore structure continues to improve over a much longer period owing to the sustained pozzolanic reaction of fly ash. After approximately 360 days, A3 becomes the least porous mixture and consistently maintains lower porosity than A2 until the end of the investigation. These results suggest that, within the replacement levels considered in the present study, increasing the fly ash content from 20% to 30% provides additional long-term refinement of the pore structure, consistent with previous observations reported by Khan et al. [42].

Slag-modified concrete (A4) also exhibits lower porosity than the control mixture throughout the exposure period; however, the magnitude of improvement remains relatively limited compared with the other supplementary cementitious materials. This behavior is consistent with the permeability results discussed previously and is primarily attributed to the relatively low slag replacement employed in the present investigation, which limits both the filler effect and the subsequent secondary hydration reaction [45].

A distinctly different evolution is observed for basalt-fiber concrete (A6). Its porosity is substantially higher than that of the control concrete at the beginning of exposure, but the difference decreases rapidly with time. After approximately 360 days, A6 exhibits lower porosity than A4, A5, and A1, indicating that the long-term influence of basalt fibers differs fundamentally from that of mineral admixtures. As hydration proceeds, the fiber–matrix interface becomes progressively denser, while the fibers effectively restrain shrinkage deformation and the propagation of microcracks. These mechanisms collectively reduce the volume of interconnected pores during long-term exposure, resulting in a continuous improvement in matrix compactness [44].

**Figure 8:**
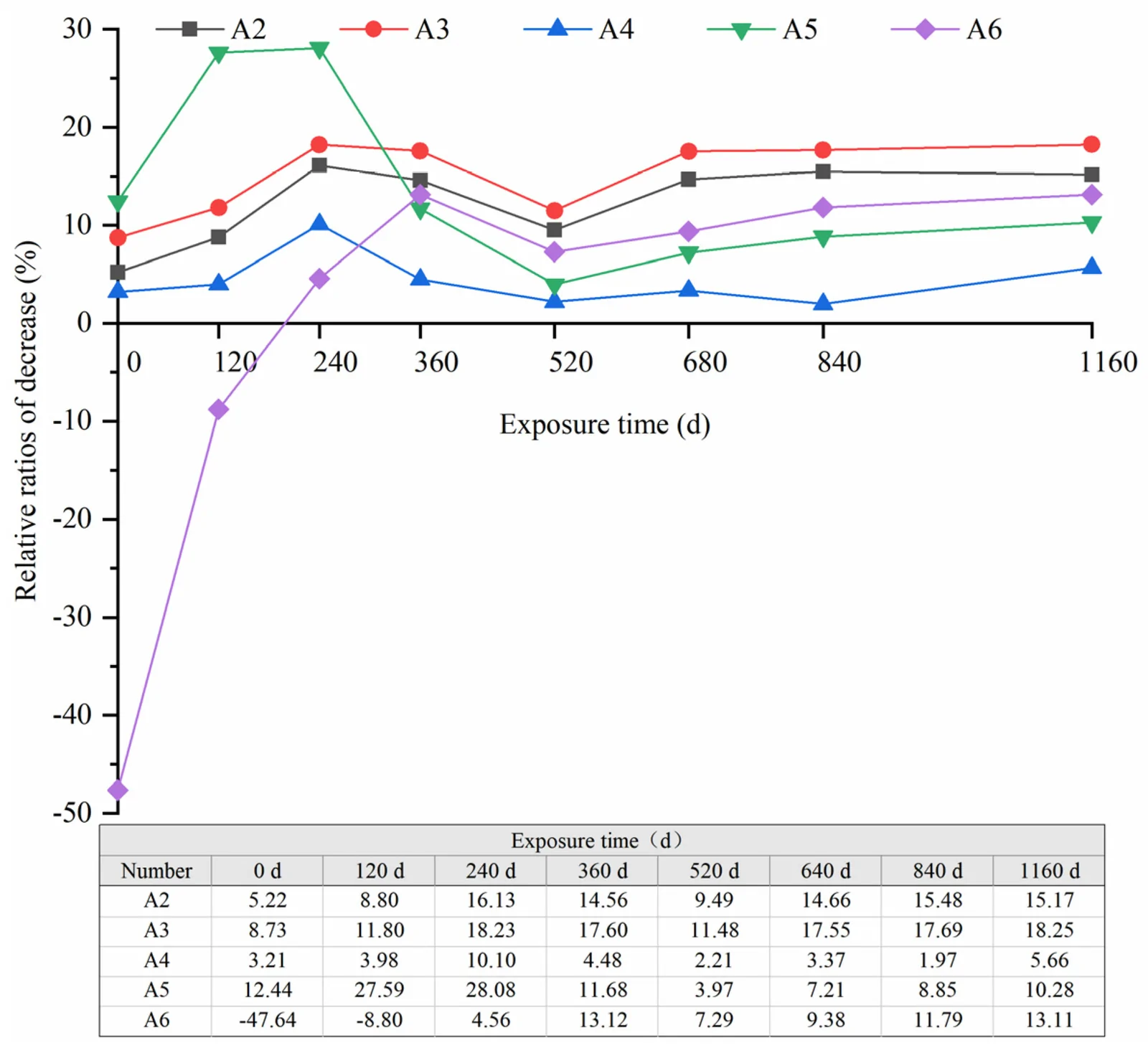
Decrease percentage at different exposure times.

To quantitatively evaluate the influence of different admixtures on porosity reduction, the porosity reduction ratio, β , is defined as

(7) $$\beta =\frac{\rho_{0}-\rho_{i}}{\rho_{0}}\times 100\%$$

where $\rho_{0}$ and $\rho_{i}$ denote the total porosity of the plain concrete and the corresponding admixture-modified concrete, respectively. The calculated values of β are illustrated in Figure 8.

As shown in Figure 8, the variation in β follows a pattern remarkably similar to that observed for the permeability reduction ratio *α* in Figure 5. Mixtures exhibiting larger reductions in total porosity generally display greater improvements in gas impermeability, indicating a strong relationship between pore volume refinement and transport resistance. Nevertheless, the correspondence is not completely identical for all mixtures, suggesting that permeability evolution is also affected by other pore characteristics, including pore size distribution and pore connectivity. Therefore, although total porosity provides an effective indicator of permeability development, it cannot fully explain the transport behavior of concrete when considered independently [24].

#### 3.3.3. Pore Size Distribution

**Figure 9:**
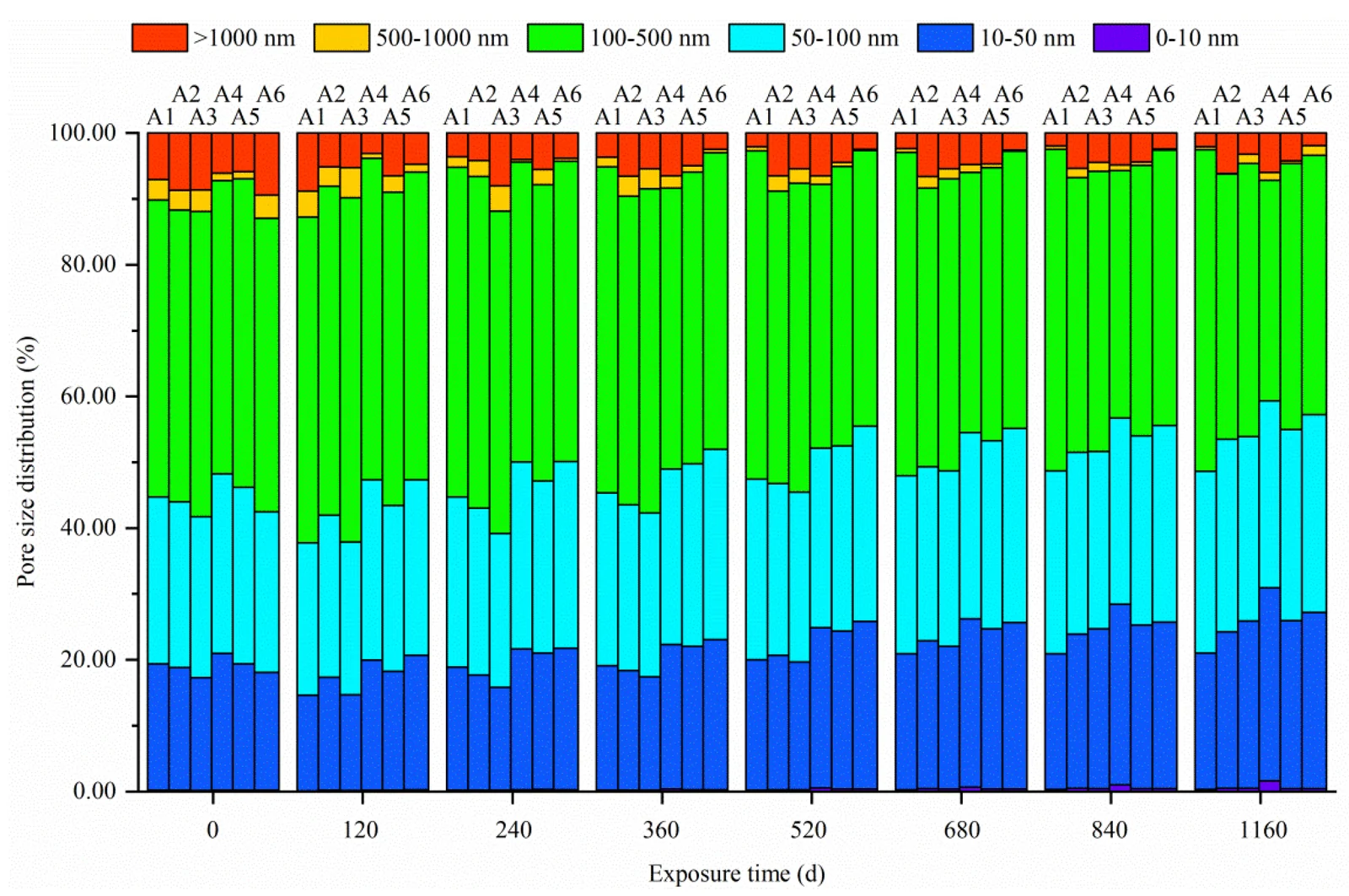
Time-varying process of pore diameter proportion of concrete.

To further elucidate the evolution of the internal pore system, the pore size distribution (PSD) derived from the NMR measurements was analyzed. Following the classification proposed in previous studies [36,46], the pore network was divided into six categories, namely gel pores (<10 nm), medium gel pores (10–50 nm), small capillary pores (50–100 nm), medium capillary pores (100–500 nm), large capillary pores (500–1000 nm), and non-capillary pores (>1000 nm). The corresponding variations in pore size distribution throughout the exposure period are presented in Figure 9.

The PSD results reveal that medium capillary pores (100–500 nm) constitute the dominant portion of the pore system for all investigated mixtures, accounting for approximately half of the total pore volume regardless of exposure age. By comparison, gel pores smaller than 10 nm occupy only a minor fraction of the overall pore structure, whereas pores larger than 500 nm remain limited throughout the entire investigation. These observations indicate that long-term changes in transport properties are primarily associated with the evolution of medium-sized capillary pores rather than with the smallest or largest pore classes.

The influence of exposure time is evident across all mixtures. As hydration progresses, the proportion of pores larger than 100 nm gradually decreases, while finer pore fractions become increasingly predominant. This tendency becomes particularly apparent after approximately 120 days, when continued hydration and secondary pozzolanic reactions promote the gradual subdivision of relatively coarse capillary pores into finer pores. Consequently, the pore network becomes progressively denser and less favorable for gas transport, which agrees well with the continuous reductions in both gas permeability (Table 3) and total porosity (Table 6) observed during long-term exposure [43,47].

Distinct pore refinement characteristics are observed for different admixtures. Compared with the plain concrete, both fly ash mixtures (A2 and A3) consistently exhibit a lower proportion of 100–500 nm pores together with a corresponding increase in pores below 100 nm. Moreover, the difference becomes increasingly pronounced with exposure time, indicating that the delayed pozzolanic reaction of fly ash continuously modifies the capillary pore system during long-term exposure. This behavior explains the sustained reduction in gas permeability discussed previously in Section 3.1.

The slag mixture (A4) exhibits a similar but less pronounced trend. Although the fraction of pores below 100 nm remains slightly higher than that of the reference concrete throughout the exposure period, the reduction in medium capillary pores is relatively limited. This observation is consistent with the comparatively small improvement in permeability and total porosity, suggesting that the low slag replacement adopted in the present study provides only moderate pore refinement.

Silica-fume concrete (A5) presents the most significant modification of pore size distribution during the early exposure stage. Before approximately 240 days, the proportion of pores below 100 nm increases markedly, accompanied by a rapid reduction in the 100–500 nm fraction. Thereafter, however, only minor changes occur in the PSD, indicating that the pore refinement process approaches completion relatively early. This evolution corresponds closely to the permeability results presented in Section 3.1, where silica fume exhibits the greatest initial improvement but only limited long-term enhancement.

Basalt-fiber concrete (A6) displays a distinctly different evolution pattern. During the initial exposure period, the proportions of pores larger than 100 nm exceed those of the plain concrete, reflecting the additional interfacial transition zones introduced by the randomly distributed fibers. As exposure continues, the fractions of medium and large capillary pores decrease steadily and eventually become lower than those of the control mixture. This evolution suggests that continued hydration gradually improves the compactness of the fiber–matrix interface while simultaneously suppressing the formation and propagation of microcracks, thereby promoting long-term pore refinement [48].

Overall, the PSD evolution demonstrates that the improvement in gas impermeability is governed not merely by a reduction in total pore volume but, more importantly, by the redistribution of pore sizes within the capillary range. Among the various pore classes, the continuous decrease in the 100–500 nm fraction appears to be most closely associated with the observed permeability reduction, indicating that this pore interval plays a dominant role in controlling gas transport during long-term tidal exposure. This inference is examined quantitatively in Section 3.4 through correlation analysis between permeability and pore structure parameters.

#### 3.3.4. Critical Pore Diameter, Median Pore Diameter and Mean Pore Diameter

**Figure 10:**
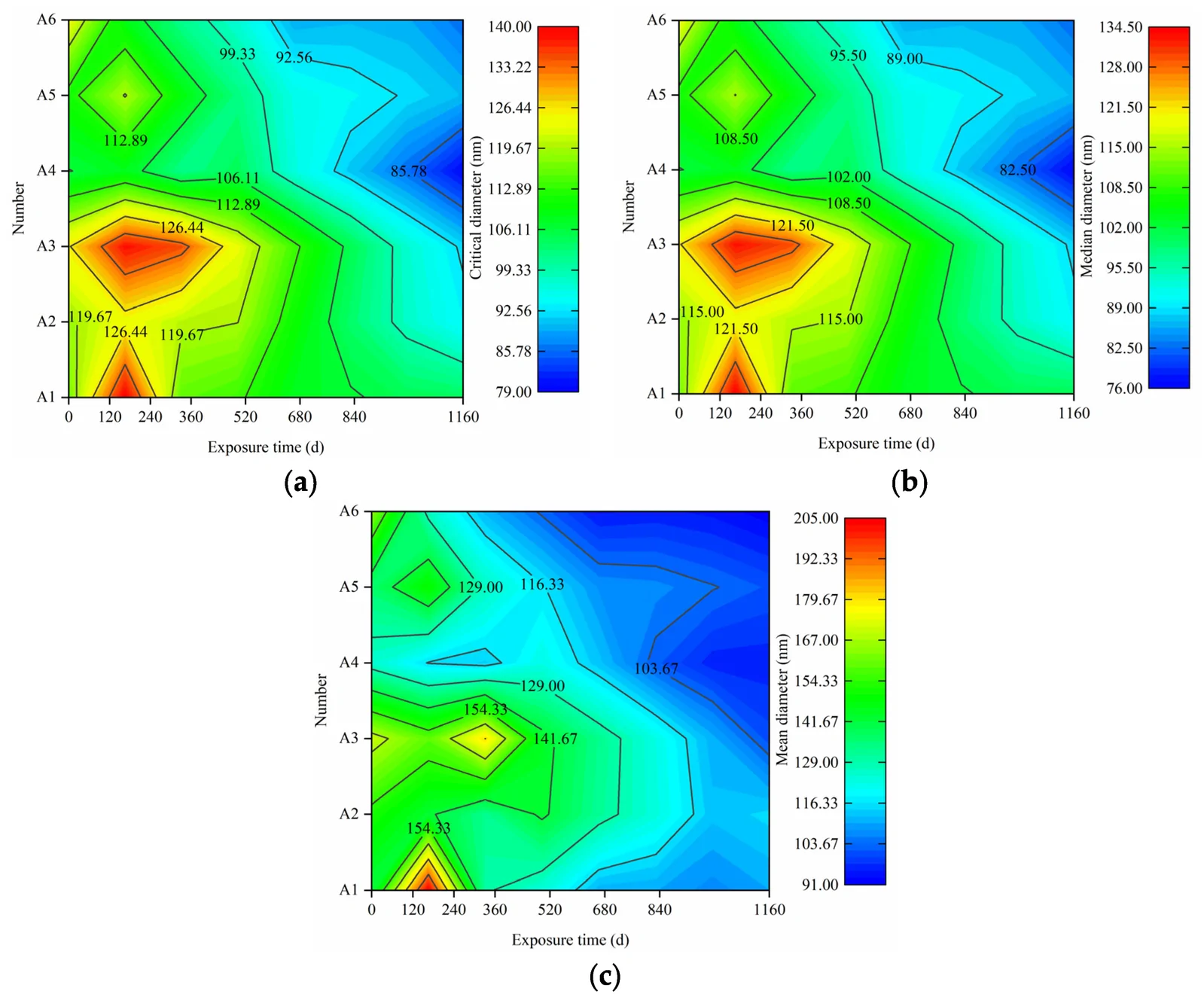
Critical pore diameter, Median pore diameter and Mean pore diameter of concrete. (**a**) Critical pore diameter of concrete *D*_cr_ at different exposure times. (**b**) Median pore diameter of concrete *D*_md_ at different exposure times. (**c**) Mean pore diameter of concrete *D*_me_ at different exposure times.

Besides total porosity and pore size distribution, characteristic pore diameters provide additional insight into the evolution of transport pathways within cementitious materials. In the present study, three representative parameters were selected for analysis, namely the critical pore diameter ( $D_{\mathrm{cr}}$ ), median pore diameter ( $D_{\mathrm{md}}$ ), and mean pore diameter ( $D_{\mathrm{me}}$ ). The critical pore diameter corresponds to the dominant peak of the pore size distribution and represents the characteristic dimension of the interconnected capillary network governing fluid transport [27]. The median pore diameter is defined as the pore size corresponding to 50% of the cumulative NMR signal, whereas the mean pore diameter is calculated as the volume-weighted average of all pores according to

(8) $${\log r}_{m}=\sum_{i}f_{i}\log r_{i}/\sum_{i}f_{i}$$

where $r_{m}$ is the average pore radius, $f_{i}$ is the volume fraction associated with pores of radius $r_{i}$ , and $r_{i}$ denotes the pore radius [46]. The variations in these three characteristic pore diameters throughout the exposure period are presented in Figure 10.

Although the three parameters exhibit different sensitivities to pore evolution, they collectively demonstrate a gradual refinement of the pore system during long-term tidal exposure. For most mixtures, both the critical and median pore diameters increase slightly during the initial 120 days before decreasing continuously at later ages. This transient increase most likely reflects the coexistence of ongoing hydration, moisture redistribution, and pore restructuring during the early exposure period. In contrast, the mean pore diameter generally exhibits a continuous downward trend throughout the investigation, indicating an overall reduction in the average pore size as hydration proceeds.

Distinct responses are observed for the different admixtures. For the fly ash concretes (A2 and A3), the mean pore diameter remains consistently larger than that of the reference concrete over the entire exposure period. Meanwhile, both the critical and median pore diameters exceed those of A1 before approximately 520 days but gradually become smaller thereafter. This evolution suggests that the delayed pozzolanic reaction of fly ash requires a relatively long period to effectively modify the interconnected capillary network. Once sufficient secondary hydration products have accumulated, the dominant transport pores are progressively refined, leading to smaller characteristic pore diameters during long-term exposure. Although A3 initially presents larger characteristic pore sizes than A2, the difference gradually diminishes with increasing exposure time, indicating that the influence of fly ash dosage becomes less significant after prolonged hydration.

The slag mixture (A4) exhibits the greatest reduction in both critical and median pore diameters during the early exposure stage. At 120 and 240 days, reductions of approximately 23% and 12%, respectively, are observed relative to the control concrete. These reductions closely correspond to the permeability improvement discussed in Section 3.1, implying that the permeability evolution of slag concrete is more closely associated with changes in the characteristic transport pore size than with variations in total pore volume alone.

Silica-fume concrete (A5) displays a different development pattern. All three characteristic pore diameters decrease mainly during the first 520 days, after which only minor changes are observed. In particular, the median pore diameter decreases by merely 1.74% between 680 and 840 days, demonstrating that the pore refinement process has largely stabilized. This tendency is consistent with the permeability evolution presented earlier, where silica fume contributes predominantly to early-age densification but provides relatively limited additional refinement during long-term exposure.

Basalt-fiber concrete (A6) again exhibits a distinctive evolution compared with the mineral admixtures. Before 120 days, all three characteristic pore diameters are larger than those of the plain concrete because the incorporation of fibers introduces additional interfacial transition zones and locally increases pore dimensions. Continued hydration progressively strengthens the fiber–matrix interface and suppresses microcrack development, leading to a continuous reduction in characteristic pore size after the initial exposure stage. Consequently, the long-term evolution of $D_{\mathrm{cr}}$ , $D_{\mathrm{md}}$ , and $D_{\mathrm{me}}$ closely follows the corresponding trends observed for permeability and total porosity, confirming the beneficial role of basalt fibers in improving the compactness of the cementitious matrix during extended exposure.

Taken together, the three characteristic pore diameters consistently indicate progressive refinement of the internal pore network throughout the tidal exposure period. Nevertheless, the evolution of the critical and median pore diameters corresponds more closely to the permeability variations than does that of the mean pore diameter. This difference suggests that gas transport is governed primarily by the characteristic dimensions of the interconnected capillary network rather than by the average size of all pores. The quantitative relationships between these pore descriptors and gas permeability are examined in detail in Section 3.4.

### 3.4. Relationship Between Gas Permeability and Microstructure Parameters

#### 3.4.1. Relationship Between Concrete Gas Permeability and Porosity

**Figure 11:**
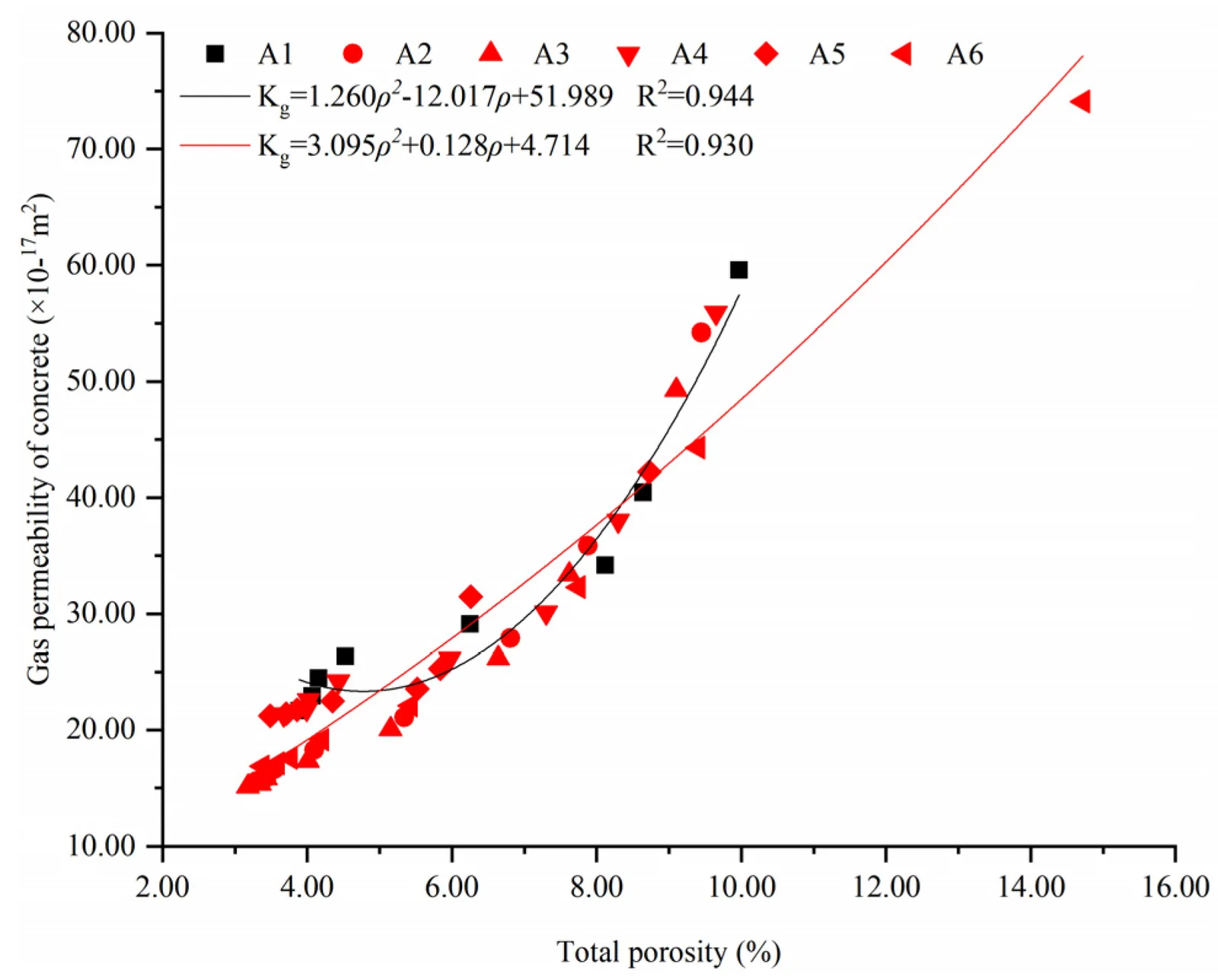
Correlation between total porosity and gas permeability of concrete.

To clarify the role of pore volume in controlling gas transport, the relationship between gas permeability and total porosity was established using the experimental results obtained at all exposure ages. The corresponding regression curves are presented in Figure 11.

The fitted relationships demonstrate that gas permeability increases consistently with total porosity for both the plain and admixture-modified concretes. Regardless of mixture composition, specimens with larger pore volumes exhibit higher permeability coefficients, confirming that pore densification remains one of the principal factors governing gas transport under long-term tidal exposure. Although the absolute permeability differs among the investigated mixtures, the overall evolution follows a similar tendency, indicating that changes in total porosity provide a reliable first-order description of permeability development.

The quality of the regression is high for every concrete mixture, with all coefficients of determination exceeding 0.90. Such strong correlations indicate that the reduction in pore volume during hydration is closely accompanied by a corresponding decline in gas permeability. Nevertheless, slight differences among the fitted curves suggest that total porosity alone cannot fully explain the transport behaviour of admixture-modified concretes.

Compared with the reference concrete (A1), mixtures incorporating supplementary cementitious materials generally exhibit slightly lower coefficients of determination. This behaviour is most likely associated with the refinement of the pore system produced by mineral admixtures. As the proportion of extremely fine pores increases, gas transport becomes increasingly influenced by the Klinkenberg (gas-slippage) effect, resulting in greater variability between total porosity and measured permeability [36,49]. Consequently, concretes possessing similar porosities may still exhibit different gas permeabilities because the connectivity and characteristic dimensions of their transport pores are not identical.

The quadratic regression equations describing the permeability–porosity relationship for each modified mixture are summarized in Table 7.

Despite the differences in regression coefficients, all fitted equations exhibit satisfactory agreement with the experimental observations, with R^2^ values ranging from 0.942 to 0.995. Among the investigated mixtures, basalt-fiber concrete (A6) produces the strongest correlation, whereas silica-fume concrete (A5) exhibits the largest scatter. These differences suggest that the influence of pore refinement on gas transport varies with admixture type. In fiber-reinforced concrete, permeability evolution appears to be governed predominantly by changes in pore volume, while in silica-fume concrete the rapid development of ultrafine pores and the associated modification of pore connectivity introduce additional complexity into the permeability response.

Overall, the regression analysis confirms that total porosity is an effective macroscopic indicator for evaluating gas permeability during long-term exposure. However, because permeability depends not only on the quantity of pores but also on their size, continuity, and connectivity, additional pore descriptors are required to establish a more comprehensive understanding of transport behaviour. Accordingly, the relationship between permeability and pore size distribution is further examined in the following section.

#### 3.4.2. Relationship Between Gas Permeability and Pore Size Distribution

While total porosity provides an overall measure of the pore volume, it does not distinguish the respective contributions of pores with different sizes to gas transport. To further identify the pore fraction primarily responsible for permeability evolution, correlation analyses were performed between the gas permeability coefficient and the volumetric proportion of each pore size interval. The corresponding coefficients of determination are summarized in Table 8.

The results indicate that the influence of pore size on gas permeability is highly size dependent. Although measurable correlations are observed for all pore classes, the strongest relationship is generally associated with pores within the 100–1000 nm range. This finding suggests that pores of intermediate capillary size constitute the principal transport pathways controlling gas flow through the concrete matrix. By contrast, the smallest gel pores contribute relatively little to permeability because their limited dimensions restrict gas movement despite their considerable abundance.

A more detailed examination of the individual pore intervals reveals that the 100–500 nm fraction exhibits the greatest sensitivity to permeability evolution for most mixtures. This pore class not only represents the largest proportion of the total pore system but also forms the most effective interconnected network for gas transmission. Consequently, reductions in this fraction during long-term exposure are accompanied by corresponding decreases in gas permeability, highlighting its dominant role in governing transport behaviour under tidal conditions.

The incorporation of supplementary cementitious materials modifies these correlations by progressively refining the capillary pore network. Compared with the plain concrete, mixtures containing fly ash, slag, or silica fume generally exhibit stronger correlations between permeability and the finer pore fractions (10–50 nm and 50–100 nm). This shift reflects the continuous transformation of medium capillary pores into finer pores through secondary hydration and pozzolanic reactions, which alters the relative contribution of each pore class to gas transport [14,16,45]. Consequently, permeability becomes increasingly influenced by changes occurring within the finer capillary range rather than solely by the presence of larger pores.

Basalt-fiber concrete displays a somewhat different behaviour. Improvements in the correlation coefficients are observed across nearly all fine-pore intervals, indicating that fiber reinforcement affects not only pore refinement but also the structural stability of the transport network. As hydration proceeds, the fibers enhance the integrity of the cementitious matrix by restraining shrinkage-induced cracking and limiting the development of new interconnected pathways [41]. As a result, the evolution of permeability is more consistently linked with the redistribution of fine pores than in the plain concrete.

Although pores exceeding 1000 nm exhibit relatively high correlation coefficients in several individual mixtures, their volumetric proportion remains very small throughout the exposure period. Therefore, their contribution to the overall permeability evolution is considered secondary compared with that of the much more abundant 100–500 nm pores. Similarly, pores smaller than 10 nm occupy only a limited transport function because most of these pores are isolated within the hydration products and do not participate directly in continuous gas flow.

Taken together, the correlation analysis demonstrates that the evolution of gas permeability is controlled primarily by changes within the capillary pore system rather than by the entire pore population. Among all pore categories considered, the 100–500 nm fraction emerges as the most representative structural parameter for describing permeability development during long-term tidal exposure. This conclusion is consistent with the pore size evolution discussed in Section 3.3.3 and provides a mechanistic explanation for the permeability reductions observed throughout the exposure period.

#### 3.4.3. Correlation Between Gas Permeability and Characteristic Pore Diameters

**Figure 12:**
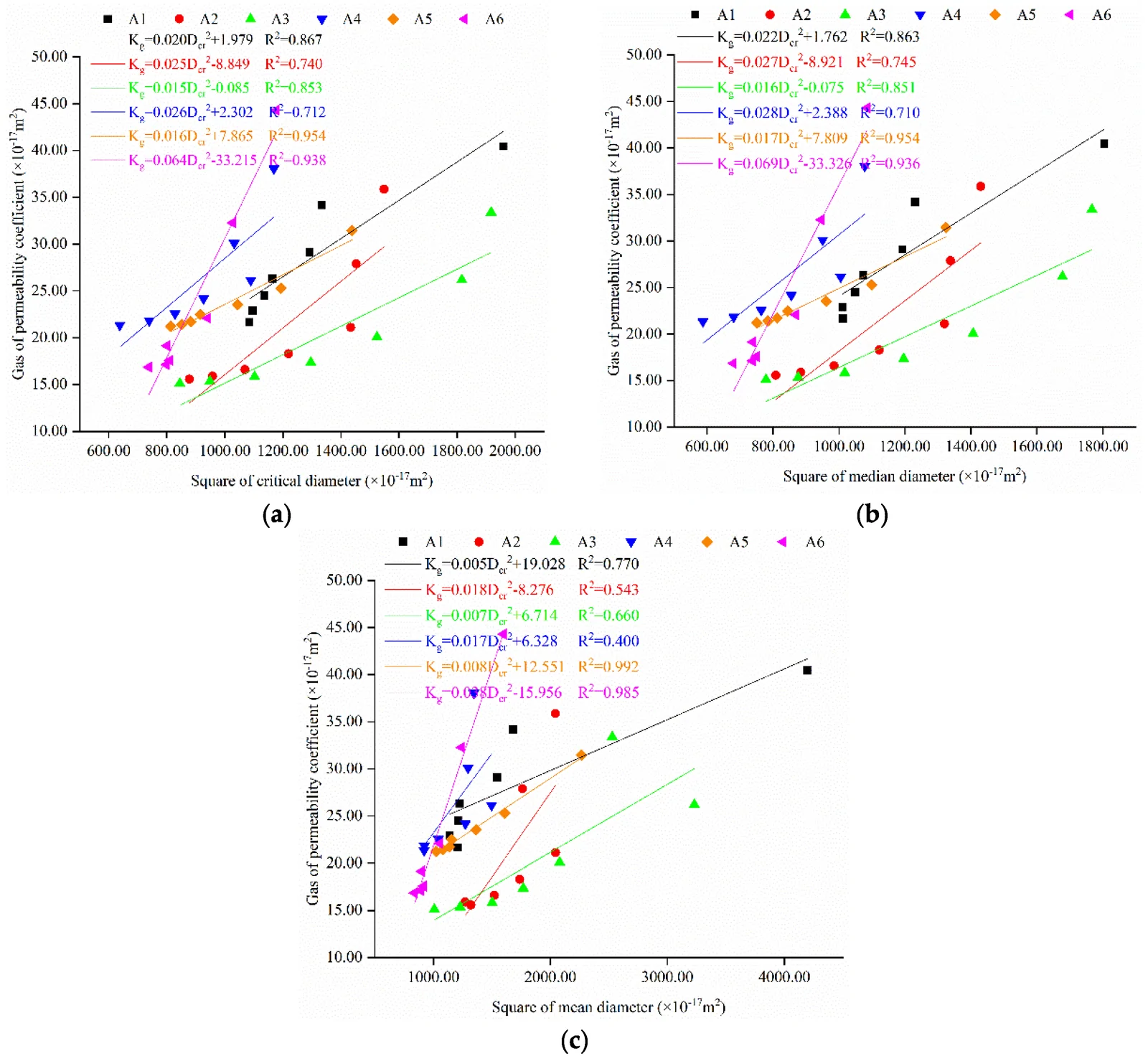
Correlation between $D_{\mathrm{cd}}^{2}$ , $D_{\mathrm{md}}^{2}$ , $D_{\mathrm{me}}^{2}$ and $K_{g}$ of concrete. (**a**) Relation between $D_{\mathrm{cr}}^{2}$ and $K_{g}$ . (**b**) Relation between $D_{\mathrm{md}}^{2}$ and $K_{g}$ . (**c**) Relation between $D_{\mathrm{me}}^{2}$ and $K_{g}$ .

To further clarify which characteristic pore parameter best represents gas transport behavior, the relationships between gas permeability and the squared values of the critical pore diameter ( $D_{\mathrm{cr}}^{2}$ ), median pore diameter ( $D_{\mathrm{md}}^{2}$ ), and mean pore diameter ( $D_{\mathrm{me}}^{2}$ ) were evaluated according to the concepts proposed by Katz–Thompson [28,50] and Garboczi [29]. The corresponding regression results are presented in Figure 12.

For all investigated mixtures, gas permeability increases with increasing characteristic pore diameter, indicating that larger transport pores facilitate the formation of continuous pathways for gas flow. This overall tendency is consistent with the progressive refinement of the pore system observed during long-term tidal exposure and further supports the close dependence of permeability on the geometry of the capillary network rather than solely on the total pore volume.

Although all three characteristic diameters exhibit positive correlations with permeability, their predictive capabilities differ substantially. In general, the critical pore diameter and the median pore diameter provide considerably stronger correlations than the mean pore diameter. This distinction reflects the different physical significance of these parameters. The critical pore diameter characterizes the dominant interconnected capillary pathways responsible for fluid transport, whereas the median pore diameter represents the overall distribution of effective transport pores. By comparison, the mean pore diameter incorporates contributions from the entire pore population, including numerous isolated gel pores that participate little in gas migration. Consequently, its ability to characterize permeability is inherently weaker.

For the plain concrete together with the fly ash- and slag-modified mixtures, the regression coefficients associated with $D_{\mathrm{cr}}^{2}$ and $D_{\mathrm{md}}^{2}$ generally fall within the range of approximately 0.70–0.85. Although these correlations remain satisfactory, a certain degree of scatter is evident. Such variability is expected because gas permeability is influenced not only by pore dimensions but also by pore connectivity, moisture redistribution during cyclic wetting and drying, continuing secondary hydration, and gas-slippage effects within fine pores. The combined influence of these mechanisms introduces unavoidable dispersion into the regression analysis.

A different trend is observed for silica-fume concrete (A5) and basalt-fiber concrete (A6), where the coefficients of determination exceed approximately 0.93. The stronger correlations indicate that permeability evolution in these mixtures is more directly governed by changes in the characteristic transport pore size. In silica-fume concrete, the rapid filling of capillary pores and the densification of the interfacial transition zone generate a relatively homogeneous pore system. For basalt-fiber concrete, the restraint of shrinkage-induced microcracking and the gradual improvement of the fiber–matrix interface contribute to a more stable transport network during prolonged exposure [41]. As a consequence, characteristic pore diameters provide a more representative description of permeability development for these two mixtures.

The performance of the mean pore diameter differs noticeably from that of the other two parameters. In most cases, the regression based on $D_{\mathrm{me}}^{2}$ yields lower coefficients of determination, with several values falling below 0.70. This comparatively poor performance is attributed to the averaging process inherent in the definition of $D_{\mathrm{me}}$ , which reduces its sensitivity to the evolution of the interconnected capillary system. Since numerous fine gel pores contribute substantially to the calculated mean diameter while contributing minimally to gas transport, changes in $D_{\mathrm{me}}$ do not necessarily correspond to equivalent changes in permeability.

Overall, the present results demonstrate that characteristic pore diameters associated with the effective transport network provide more reliable predictors of gas permeability than parameters representing the average pore system. Among the three descriptors examined, the critical pore diameter and the median pore diameter exhibit the strongest and most consistent relationships with permeability, indicating that they are better suited for evaluating transport performance under long-term tidal exposure. Combined with the analyses presented in Section 3.4.1 and Section 3.4.2, these findings confirm that the evolution of gas permeability is governed by the coupled effects of pore volume reduction, capillary pore refinement, and the progressive modification of the interconnected pore network, rather than by any single microstructural parameter alone.

## 4. Conclusions

Based on the experimental results and analysis, the following conclusions can be drawn.

(1) Admixtures have different effects on reducing the gas permeability coefficient of concrete at different exposure times. SF has the strongest reduction effect before 240 d. FA shows the strongest reduction effect after 360 d. BF has an adverse effect before 120 d, but its ability to reduce gas permeability becomes evident after 240 d. Among the admixtures tested, slag has the weakest effect on reducing the gas permeability coefficient.
(2) The pore-refining effects of the admixtures differ. With increasing exposure time, the ability of admixtures to improve pore size distribution gradually becomes apparent. Among them, SG has the most obvious effect on reducing the critical pore diameter and median pore diameter.
(3) The addition of admixtures affects the correlations between gas permeability coefficient, total porosity and the proportions of the main pore-size ranges. The time-dependent nitrogen permeability coefficient of concrete is most sensitive to the proportion of pores in the 100–500 nm range.
(4) Adding FA and SG decreases the correlations between gas permeability coefficient and the critical, median and mean pore diameters, whereas adding SF and BF improves these correlations. Compared with mean pore diameter, critical pore diameter and median pore diameter have stronger influences on the gas permeability coefficient of concrete.

## Figures and Tables

**Table 1:** Chemical compositions of cement and various admixtures (%).

Designation	SiO_2_	Al_2_O_3_	Fe_2_O_3_	CaO	MgO	SO_3_	K_2_O	TiO_2_
Cement	24.55	10.48	2.16	51.16	6.01	2.78	—	—
FA	47.66	20.81	9.84	11.51	1.51	—	1.65	—
SG	36.38	17.60	0.89	28.18	14.19	—	—	0.74
SF	98.02	0.42	0.09	0.29	0.41	0.31	—	—
BF	54.00	16.50	11.50	7.00	4.50	—	4.50	1.50

**Table 2:** Mixture proportions of test concrete and corresponding 28 d compressive strength.

No.	Cement	Water	Sand	Gravel	Admixture		28 d Compressive Strength (MPa)
	(kg/m^3^)				Type	Content (kg/m^3^)	
A1	380	190	578	1229	—	—	25.7
A2	304	190	578	1229	FA	76	22.2
A3	266	190	578	1229	FA	114	21.2
A4	361	190	578	1229	SG	19	28.6
A5	361	190	578	1229	SF	19	31
A6	380	190	578	1229	BF	1.026	29.3

**Table 3:** Average value of gas permeability coefficient (×10^−17^ m^2^).

No.	Exposure Time (d)							
	0	120	240	360	520	680	840	1160
A1	59.59	40.46	34.18	29.11	26.32	24.48	22.91	21.66
A2	54.22	35.86	27.90	21.10	18.29	16.59	15.89	15.58
A3	49.29	33.38	26.18	20.06	17.35	15.84	15.35	15.12
A4	55.91	38.04	30.10	26.15	24.18	22.56	21.83	21.35
A5	42.22	31.45	25.30	23.53	22.49	21.72	21.41	21.22
A6	74.09	44.30	32.28	22.08	19.15	17.59	17.12	16.86

**Table 4:** Age reduction factor of concrete gas permeability coefficient.

Exposure Time (d)	No.					
	A1 (R^2^)	A2 (R^2^)	A3 (R^2^)	A4 (R^2^)	A5 (R^2^)	A6 (R^2^)
0–360 d	0.253 (0.819)	0.304(0.792)	0.289(0.795)	0.268 (0.814)	0.212(0.824)	0.379(0.773)
0–520 d	0.259 (0.854)	0.320(0.823)	0.306 (0.821)	0.272 (0.849)	0.212(0.859)	0.397 (0.805)
0–680 d	0.264(0.874)	0.329 (0.843)	0.315 (0.844)	0.274(0.871)	0.211 (0.879)	0.405 (0.827)
0–840 d	0.267 (0.888)	0.334 (0.859)	0.319 (0.860)	0.274 (0.886)	0.208 (0.892)	0.409 (0.842)
0–1160 d	0.266(0.902)	0.334(0.871)	0.319(0.873)	0.271(0.896)	0.203(0.896)	0.407(0.855)

**Table 5:** Predicted value and relative error of gas permeability coefficient of concrete at 1160 d.

No.	A1	A2	A3	A4	A5	A6
Measured value (×10^−17^ m^2^)	21.66	15.58	15.12	21.35	21.22	16.86
predicted value (×10^−17^ m^2^)	21.87	15.28	14.67	19.90	19.31	15.89
relative error (%)	0.97	1.93	2.98	6.79	9.00	5.75

**Table 6:** Total porosity of concrete at different exposure times (%).

No.	Exposure Time (d)							
	0	120	240	360	520	680	840	1160
A1	9.97	8.64	8.12	6.25	4.53	4.16	4.07	3.89
A2	9.45	7.88	6.81	5.34	4.10	3.55	3.44	3.30
A3	9.10	7.62	6.64	5.15	4.01	3.43	3.35	3.18
A4	9.65	8.30	7.30	5.97	4.43	4.02	3.99	3.67
A5	8.73	6.26	5.84	5.52	4.34	3.86	3.71	3.49
A6	14.72	9.40	7.75	5.43	4.20	3.77	3.59	3.38

**Table 7:** Correlation between total porosity and gas permeability of concrete with admixtures.

No.	Fitting Formula	R^2^
A2	$K_{g}=1.000\rho^{2}-6.455\rho +27.665$	0.982
A3	$K_{g}=0.965\rho^{2}-6.083\rho +25.980$	0.986
A4	$K_{g}=1.262\rho^{2}-11.511\rho +48.233$	0.977
A5	$K_{g}=0.595\rho^{2}-3.177\rho +24.927$	0.942
A6	$K_{g}=0.160\rho^{2}+2.250\rho +6.746$	0.995

**Table 8:** Relationship between pore size contribution and gas permeability coefficient of concrete.

No.		A1	A2	A3	A4	A5	A6
Pore size interval (nm)	0–10	0.861	0.731	0.803	0.660	0.854	0.742
	10–50	0.771	0.868	0.864	0.865	0.977	0.939
	50–100	0.834	0.673	0.932	0.958	0.969	0.988
	100–500	0.982	0.953	0.852	0.715	0.968	0.805
	500–1000	0.805	0.546	0.986	0.165	0.902	0.866
	<100	0.811	0.825	0.892	0.810	0.983	0.968
	100–1000	0.921	0.905	0.900	0.907	0.975	0.909
	>1000	0.798	0.743	0.585	0.430	0.970	0.992

## Data Availability

The original contributions presented in the study are included in the article. Further inquiries can be directed to the corresponding author.
