# Correction: Wang et al. Effect of Admixtures on Time-Dependent Gas Permeability of Concrete and Correlation with Its Microstructure Parameters. *Materials* 2026, *19*, 3389

**DOI:** 10.3390/ma19173716

**Published:** 2026-08-31

**Authors:** Jiandong Wang, Jingzong Xu, Shumeng Zhang, Yinghui Cao

**Affiliations:** 1 Campus Construction Office, Zhejiang University of Technology, Hangzhou 310014, China; wjd@zjut.edu.cn; 2 College of Civil Engineering, Zhejiang University of Technology, Hangzhou 310014, China; 221125060089@zjut.edu.cn (J.X.); 302024524033@zjut.edu.cn (S.Z.)

In the original publication [1], there was an error regarding the affiliation of Jiandong Wang and Yinghui Cao. Affiliation 1 “Campus Construction Office, Zhejiang University of Technology, Hangzhou 310014, China” was not included.

The authors state that the scientific conclusions are unaffected. This correction was approved by the Academic Editor. The original publication has also been updated.

## References

[1] Wang J., Xu J., Zhang S., Cao Y. (2026). Effect of Admixtures on Time-Dependent Gas Permeability of Concrete and Correlation with Its Microstructure Parameters. Materials.

